# Evaluation of the effectiveness of psychosocial treatment of patients with schizophrenia at different stages of its rendering

**DOI:** 10.1192/j.eurpsy.2023.2193

**Published:** 2023-07-19

**Authors:** V. Mitikhin, G. ?. Tiumenkova, T. Solokhina, L. Alieva

**Affiliations:** 1 Department of Mental Health Support Systems Research Centre, Mental Health Research Centre, Moscow, Russian Federation

## Abstract

**Introduction:**

Currently, there is an active introduction of modern types of psychosocial treatment (PST). At the same time, an important direction is the evaluation of the effectiveness of PST, the identification of factors affecting it, which determines the urgency of research in this area.

**Objectives:**

To evaluate the effectiveness of psychosocial treatment of patients with schizophrenia at different stages of psychiatric care; to build regression models to identify factors that influence the effectiveness of psychosocial treatment.

**Methods:**

Clinical and psychopathological, statistical, as well as a battery of tests: PANSS, CGI; Drug Attitude Inventory (DAI, Hogan T.P. et al., 1983); Insight Scale for Psychosis (ISP, Birchwood M., 1994); «SF-36 Health Status Survey» (SF-36, Ware J.E. et al., 1993); URICA (McConnaughy E.A. et al., 1983); The Social Adjustment Scale-Self (SAS-SR, Weissman M, Bothwell S.,1976); PSP (Morosini P.L. et al., 2000) and a number of other scales. 90 patients with schizophrenia in the community, inpatient department of psychiatric hospital, day hospital participated in the basic PST program, which included psychoeducation, motivational training, social and cognitive skills training. At each stage, PST was received by 30 patients who did not differ significantly in age and other socio-demographic characteristics, but were characterized by different quality of remission, the duration of the PST program was 3 months. The assessment of the patients’ condition was carried out before and after the rehabilitation program.

**Results:**

A significant improvement in the indicators on the PANSS scale was found in patients of all three groups, as evidenced by a reduction of more than 10 points in the total score of the scale. As a result of the PST program, patients of all three groups have improved to varying degrees their awareness of the disease understanding of the need for drug treatment, increased motivation, and have shown a tendency to improve a number of cognitive functions. The participants of the program demonstrated an increase in the level of activity and purposefulness of activity, as well as the ability to master new social skills and implement them. Correlation and regression analysis, during which more than 100 factors were studied, allowed us to identify the most significant factors that positively or negatively affected the effectiveness of PST: severity of the condition, duration of illness, age of onset of the disease, age of referral to the service, number of hospitalizations, type of remission, observation group, level of education, marital status, family support, family relationships, having friends, having income.

**Conclusions:**

The effectiveness of the basic PST program has been shown. However, the work on evaluating the effectiveness of the PST should be continued, especially for the development of information criteria and a tool for its evaluation.

**Disclosure of Interest:**

None Declared

